# Intralobar pulmonary sequestration with cystic degeneration mimicking a bronchogenic cyst in an elderly patient: A case report and literature review

**DOI:** 10.1097/MD.0000000000019347

**Published:** 2020-02-28

**Authors:** Han Joon Kim, Kyung Eun Shin, Jai Soung Park, Heon Lee, Jae Wook Lee, Susie Chin, Hwa Kyun Shin

**Affiliations:** a Department of Radiology; b Department of Pathology; c Department of Thoracic and Vascular Surgery, Soonchunhyang University Hospital Bucheon, Bucheon, South Korea.

**Keywords:** bronchogenic cyst, pulmonary sequestration, computed tomography, magnetic resonance imaging

## Abstract

**Introduction:**

Pulmonary sequestration (PS) is a rare congenital malformation defined as nonfunctioning lung tissue supplied by systemic circulation. It is uncommonly diagnosed in adults. Herein, we describe a clinical case of PS with cystic degeneration mimicking a bronchogenic cyst in an elderly patient.

**Patient concerns:**

A huge cystic mass was incidentally found in a 65-year-old man on chest computed tomography (CT) scans during preoperative workup for a hand laceration. A 15-cm-sized round cystic mass was detected in the right lower lobe.

**Diagnosis:**

After reviewing the chest CT scan, we decided to perform contrast-enhanced chest magnetic resonance imaging (MRI) and CT-guided lung aspiration biopsy. On MRI, the lesion had the appearance of a cystic mass with hemorrhagic clots, such as an intrapulmonary bronchogenic cyst. The aspirated specimen was nondiagnostic; thus, we decided to surgically remove the mass.

**Interventions:**

Upon right lower lobectomy, the mass was diagnosed as a PS. A thin systemic artery supplying the cystic mass was visualized during surgery.

**Outcomes:**

The patient is undergoing regular follow-up at the outpatient clinic.

**Conclusions:**

PS should be considered as a differential diagnosis in patients with a cystic lung mass. Identification of a systemic artery on radiologic imaging is important in the diagnosis of PS before preoperative workup to prevent unpredicted massive bleeding during surgery.

## 1 Introduction

Pulmonary sequestration (PS) is a rare congenital malformation,^[1]^ defined as nonfunctioning lung tissue supplied by one or more systemic arteries with no direct connection to the normal tracheobronchial tree.^[1]^ Because most patients with PS are diagnosed early in life, it is considered a childhood disease.^[1,2]^ However, it is occasionally found in other age groups, with or without symptoms, and imaging findings on computed tomography (CT) and magnetic resonance imaging (MRI) are variable due to inflammation and infection.^[2]^ Thus, PS is often misdiagnosed as another respiratory disease, such as pneumonia, lung cancer, mediastinal tumor, or lung cysts in adults.^[3]^ This case report is an example of a PS mimicking a bronchogenic cyst in a 65-year-old male.

## 2 Case report

**Figure 1:**
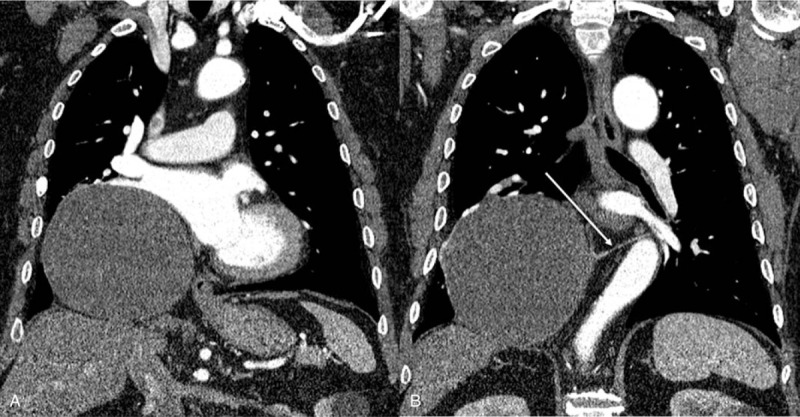
(A and B) Contrast-enhanced chest computed tomography (CT) shows a huge cystic mass measuring 15 cm in the right lower lobe. There is a thin aberrant systemic artery from the descending thoracic aorta (arrow).

Patient has provided informed consent for publication of the case. A 65-year-old male visited our hospital due to lacerations to both hands while using an automated cutting machine. During preoperative workup, a huge mass was found in the right lower lobe on chest radiograph. He had a history of hypertension, diabetes mellitus, and decreased renal function. He had never smoked, and had no fever or abnormal respiratory symptoms such as dyspnea, cough, or sputum. Laboratory studies revealed the following: white blood cell count, 71,100/μL; hemoglobin, 12.5 g/dL; platelet count, 239,000/μL; erythrocyte sedimentation rate, 33 mm/hour; and C-reactive protein, 0.83 mg/dL. The tumor markers showed a carcinoembryonic antigen level of 4.73 ng/mL (normal range: <4.7 ng/mL). To evaluate the lung mass, contrast-enhanced chest CT was performed. The chest CT revealed a 15-cm, well-marginated huge mass in the right lower lobe. The mass was located in the right cardiophrenic angle, showing a mass effect on the right inferior pulmonary vein, left atrium, and right lower lobe. By measuring the region of interest, the attenuation of the mass was found to be about 28∼30 Hounsfield units on unenhanced CT and there was no significant net enhancement on contrast-enhanced CT. Therefore, the mass was suspected to be a cystic mass, such as a bronchogenic cyst (Fig. 1A and B).

**Figure 2:**
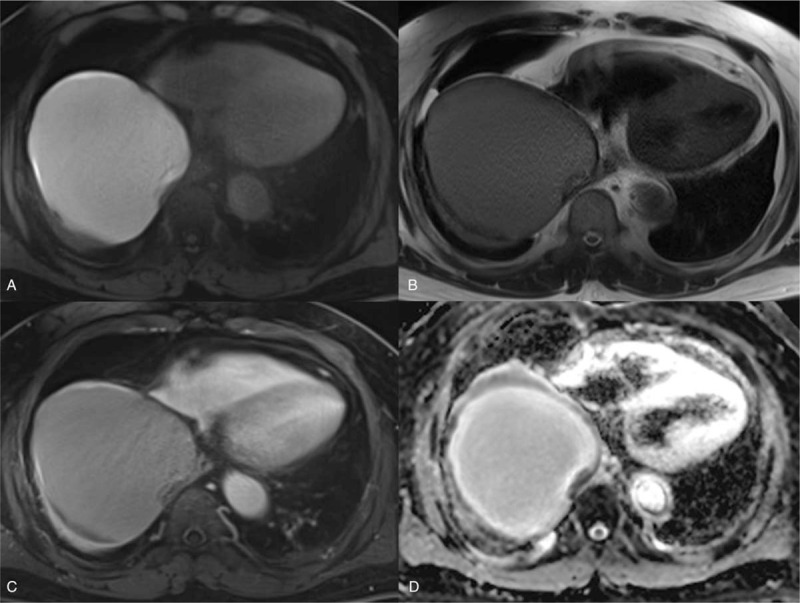
(A–D) T1-weighted thoracic MRI, T2-weighted thoracic MRI, T1-weighted enhanced thoracic MRI, and ADC map. The huge mass shows higher signal intensity than CSF on T1-weighted images (A) and an intermediate-to-low signal intensity compared to CSF on T2-weighted images (B), suggestive of a cystic lesion with subacute stage hemorrhage. T1-weighted enhanced MRI (C) reveals mild irregular rim enhancement of the cystic wall with blood clots. The ADC map (D) shows high signal intensity, suggesting the cystic nature of the mass. ADC = apparent diffusion coefficient, CSF = cerebrospinal fluid, MRI = magnetic resonance image.

CT-guided aspiration was recommended for the cystic lesion. However, the cystic lesion was too sticky to provide sufficient aspirated specimens. Contrast-enhanced chest MRI was then performed for further evaluation. The MRI showed signal intensity higher than cerebrospinal fluid (CSF) on T1-weighted images (Fig. 2A). On T2-weighted images, it showed an intermediate-to-low signal intensity compared to CSF (Fig. 2B). Moreover, there were a few irregular dark nodules along the cystic wall of the lesion. On contrast-enhanced sequencing, the lesion showed mild irregular wall rim enhancement (Fig. 2C). Finally, diffusion-weighted imaging showed a benign cystic mass without diffusion restriction (Fig. 2D). Considering the CT and MRI findings, a bronchogenic cyst with hemorrhage and blood clots was the most probable diagnosis.

**Figure 3:**
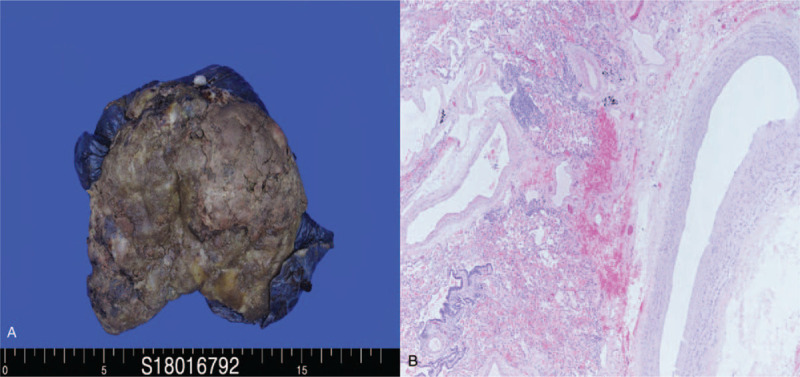
(A) The gross PS specimen. The intralobar PS shows a bluish huge mass with chronic inflammation, fibrosis, and cystic degeneration similar to an abscess. (B) Microscopic findings of the aberrant systemic artery with an elastic layer (hematoxylin and eosin stain, 40×). PS = pulmonary sequestration.

The patient underwent a video-assisted thoracoscopic excision biopsy for the cystic mass. It was impossible to isolate the mass because it was located within the parenchyma of the right lower lobe. Therefore, a right lower lobectomy was performed. A systemic artery supplying the cystic mass was observed during the procedure. The pathologic report revealed intralobar PS with chronic inflammation, fibrosis, and cystic degeneration. In addition, the systemic supplying artery was confirmed on the pathologic specimen (Fig. 3).

A review of the CT images revealed fine arterial enhancement of the systemic artery from the descending thoracic aorta.

The patient is being followed up regularly at the outpatient clinic without any remarkable symptoms or signs other than postoperative pleural effusion in the right hemithorax on chest radiograph.

## 3 Discussion

PS, also known as bronchopulmonary sequestration, is a very rare congenital pulmonary anomaly accounting for 0.15% to 6.45% of bronchopulmonary malformations.^[4]^ It consists of a nonfunctioning mass of lung tissue in the tracheobronchial tree.^[2]^ The 2 most important characteristics of PS are as follows. First, it has no direct connection to the normal bronchial tree.^[2]^ Second, it is supplied by systemic circulation. Therefore, identification of a systemic arterial supply is important for diagnosing PS.^[5]^ The definite diagnosis of PS is made by histopathologic examination after surgery; however, PS can be confirmed by contrast-enhanced CT or MRI with or without angiography by identification of a systemic arterial supply noninvasively.^[6]^ The classification of PS is based on its relation to normal lung tissue. Intralobar sequestration (ILS) occurs within normal functioning lung tissue, whereas extralobar sequestration (ELS) is completely separated from normal tissue by its own visceral pleura.^[2,3]^ There have been some studies presenting prenatally diagnosed cases of ILS,^[7]^ which can be explained by the single embryonic hypothesis, which has the common theme of airway maldevelopment originating before separation of the systemic and pulmonary circulation.^[8]^ However, most cases of ILS are late onset, so it is impossible to explain the pathogenesis of PS by the single embryonic hypothesis alone. Therefore, some researchers have insisted that the majority of ILS are probably acquired lesions caused by chronic pulmonary infection or inflammation.^[7]^

A Chinese study by Wei and Li^[3]^ that included 2625 cases showed that PS was about 1.6 times more common in male than female patients, ILS (83.95%) was about 4 times more common than ELS (16.05%), and the ILS/ELS ratio was higher in the adult group than in the pediatric group. However, ILS (20 ± 8 years) was diagnosed at an earlier age than ELS (38 ± 9 years) because ILS is usually symptomatic due to recurrent infection or inflammation.

The CT features of PS are variable, and in an analysis of 2625 Chinese cases the most common CT findings were mass lesions, followed by cystic lesions, cavitary lesions, pneumonic lesions, and bronchiectasis.^[3]^ Because of the diversity of possible CT findings, 58.63% of the cases in that study were incorrectly diagnosed before surgery. However, the study was based on a retrospective analysis of data contained within the National Knowledge Infrastructure, where images were interpreted by a diverse group that were not part of the research team, and no detailed descriptions of the images were provided,.

According to radiologic studies, PS typically appears on CT as a solid mass or consolidation with homogenous or heterogenous enhancement. Less frequent features include a collection of many small cystic lesions containing air or fluid, a large cavitary lesion with an air-fluid level, or a well-defined cystic mass, as in our case.^[7]^ Cystic lesions are multicystic and 26% of cases have an air-fluid level caused by fistulous bronchial communication^[7,9]^ In addition, emphysematous changes or hyperlucency at the margin of the lesion are characteristic findings of PS resulting from collateral air drift and air trapping due to the absence of a normal bronchial connection.^[10]^

Because PS can show several different radiologic findings, it is important to identify an aberrant systemic artery.^[11]^ The systemic artery supplying the PS usually arises from the lower thoracic aorta.^[3]^ However, it can also arise from the upper abdominal aorta, and rarely from other systemic arteries such as the internal mammary artery, celiac artery, splenic artery, or even a coronary artery.^[7]^ The diameter of systemic arteries can vary from 3 to 14 mm^[5,12]^ but, as in our case, the diameter can also be less than 3 mm. Ikezoe et al^[9]^ reported a 67% demonstration rate of aberrant systemic artery in their study.

On MRI, PS usually shows a relatively high signal intensity compared to normal lung tissue on T1 and T2-weighted images; these modalities are relatively well suited for diagnosis because of their precise anatomic localization.^[13]^ However, MRI cannot delineate focal thin-walled cysts or emphysematous changes surrounding the PS.^[10]^ MRI can also be used to demonstrate aberrant artery and venous drainage, especially with enhanced three-dimensional magnetic resonance angiography.^[14,15]^ However, MRI is less feasible due to its high cost and longer scanning time.^[15]^

There is a heterogeneous group of congenital cystic lesions in adult patients, comprising congenital cystic adenomatoid malformation, bronchogenic cyst, and PS.^[16]^ In the case of a huge cystic mass without an air-fluid level, an intrapulmonary bronchogenic cyst (IBC) can be a possible differential diagnosis and should be considered as such in patients with a cystic lung mass. On CT, IBC usually appears as a well-defined spherical or oval-shaped cyst with fluid attenuation or a cyst with an air-component or air-fluid level.^[16–18]^ It can occasionally show soft tissue attenuation on CT depending on its fluid composition, such as hemorrhage or calcium material.^[16,17,19]^ Moreover, IBC sometimes has thick walls after an infection or inflammation. On MRI, it usually shows isosignal intensity compared to skeletal muscle on T1-weighted images, and a high signal intensity compared to CSF on T2-weighted images,^[20]^ but it may also show variable signal intensity on T1-weighted images.^[17]^ Thus, it is difficult to differentiate PS from IBC by other imaging features alone, except for the lack of a systemic arterial supply to the lung in IBC. So identification of the aberrant systemic artery on imaging studies using contrast enhanced chest CT or MRI is important for a preoperative provisional diagnosis of PS.

To the best of our knowledge, there are a limited number of case reports on ILS in elderly patients (aged >60 years) in the English literature (Table 1). There were 7 cases of ILS in elderly patients including the current case, and their ages ranged from 60 to 79 years. Only 2 cases were asymptomatic: one was a 60-year-old male with multiple air-filled cysts in the right lower lobe and the other is the present case. Our case is the first report on ILS found incidentally as a huge cystic mass in an elderly patient. The mass was huge, with multistage hemorrhage and blood clots. There were no emphysematous changes surrounding the normal lung and the diameter of the aberrant systemic artery was too thin to be recognizable, which made it difficult to diagnose PS by radiologic findings alone.

**Table 1:**
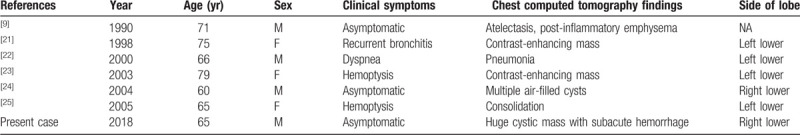
Summary of published cases of intralobar sequestration in elderly patients (>60 years old).

PS is a benign condition, but it can be accompanied by serious complications such as recurrent respiratory infection, massive hemoptysis, and heart failure.^[11]^ Therefore, the treatment for symptomatic patients with PS has always been surgical resection. Surgical resection should be considered even in asymptomatic patients with PS.^[11]^ The extent of resection depends on the subtype of PS^[26]^; the treatment of choice is mass resection for ELS and lobectomy for ILS. Because of inflammation and fibrosis, the aberrant systemic artery is difficult to dissect and bleeds easily. Therefore, during surgery, identification and ligation of all vascular connection to the PS is important. In addition, preoperative embolization may be an effective procedure by identifying all systemic vascular supply and reducing systemic arterial pressure.^[27,28]^

In conclusion, ILS manifesting as a huge cystic mass can be found in asymptomatic elderly patients. Though definite diagnosis of PS can be made through pathologic examination after surgical resection, a provisional diagnosis of PS can be made through contrast-enhanced CT or MRI by finding a systemic arterial supply. Identification of the aberrant systemic artery on preoperative imaging studies is also important for preventing bleeding during surgery.

## Author contributions

**Conceptualization:** Kyung Eun Shin.

**Resources:** Susie Chin, Hwa Kyun Shin.

**Supervision:** Kyung Eun Shin, Jai Soung Park.

**Validation:** Jae Wook Lee.

**Writing – original draft:** Han Joon Kim.

**Writing – review & editing:** Han Joon Kim, Kyung Eun Shin, Heon Lee.
